# Well-Wrapped Li-Rich Layered Cathodes by Reduced Graphene Oxide towards High-Performance Li-Ion Batteries

**DOI:** 10.3390/molecules24091680

**Published:** 2019-04-30

**Authors:** Di Liu, Fengying Wang, Gang Wang, Congjie Lv, Zeyu Wang, Xiaochuan Duan, Xin Li

**Affiliations:** 1 Department of Physics and Electronic Information Engineering, Qinghai Nationalities University, No.3 Bayizhonglu, Chengdong District, Xining 810007, China; andi1994602@163.com (D.L.); wangfy09@hotmail.com (F.W.); 2 Pen-Tung Sah Institute of Micro-Nano Science and Technology, Xiamen University, Xiamen 361005, China; 33520161152638@stu.xmu.edu.cn (C.L.); 33520171152296@stu.xmu.edu.cn (Z.W.); xcduan@xmu.edu.cn (X.D.)

**Keywords:** layered lithium-rich manganese oxide, cathode material, co-precipitation, coating

## Abstract

Layered lithium-rich manganese oxide (LLO) cathode materials have attracted much attention for the development of high-performance lithium-ion batteries. However, they have suffered seriously from disadvantages, such as large irreversible capacity loss during the first cycle, discharge capacity decaying, and poor rate performance. Here, a novel method was developed to coat the surface of 0.4Li_2_MnO_3_∙0.6LiNi_1/3_Co_1/3_Mn_1/3_O_2_ cathode material with reduced graphene-oxide (rGO) in order to address these drawbacks, where a surfactant was used to facilitate the well-wrapping of rGO. As a result, the modified LLO (LLO@rGO) cathode exhibits superior electrochemical performance including cycling stability and rate capability compared to the pristine LLO cathode. In particular, the LLO@rGO with a 0.5% rGO content can deliver a high discharge capacity of 166.3 mAh g^−1^ at a 5C rate. The novel strategy developed here can provide a vital approach to inhibit the undesired side reactions and structural deterioration of Li-rich cathode materials, and should be greatly useful for other cathode materials to improve their electrochemical performance.

## 1. Introduction

Since Sony commercialized rechargeable batteries as lithium-ion batteries in 1991, these lithium-ion batteries have revolutionized the face of consumer electronics [1,2]. Nowadays, the electronic industry is not only confined to watches, notebooks, these small electric products, but also towards the development of the large capacity, high energy density electric vehicle industry. Therefore, as the most important part of lithium-ion batteries, cathode materials are increasingly demanded. Recently, LLO cathode materials can achieve a specific capacity of more than 200 mAh g^−1^, and their voltage stability and excellent cycle life can be expected to be candidates for providing the energy for hybrid electric vehicles and pure electric vehicles [3]. However, LLO cathode materials have always seriously suffered from disadvantages, such as large irreversible capacity loss during the first cycle, discharge capacity decaying, and poor rate performance [4,5,6]. In order to solve these problems, several strategies have been developed, including surface coating [7,8,9], metal doping [10,11,12] and morphology controlling [13,14,15]. Among these modification methods, surface coating is a very effective method to improve the electrochemical properties of materials [16]. 

By far, oxides [7], fluorides [8] and phosphates [9] has been applied for surface coating. Though the cyclic stability of the cathode materials have been improved by them, their poor electronic conductivity led to a poor rate performance. Therefore, many efforts are being tried to find a coating material with superior electronic conductivity and a stable structure.

Graphene is a two-dimensional, macromolecule carbon atom sheet with a honeycomb structure. Owing to its excellent electronic conductivity and mechanical properties, graphene can serve as an ideal conductive additive for hybrid nanostructured electrodes [17,18,19,20,21,22]. Cyclic stability and the rate performance of some metals [23,24], metal oxides [20,21,25] and electrode materials modified by graphene derivatives, have been improved to some extent. However, there is still much room for graphene derivates to further enhance those electrode materials, because of the non-uniform coating of graphene derivatives on the material surface. The non-uniform coating originates from the complicated interaction between the LLO surface and graphene derivatives, which is based on electrostatic forces, the Van der Waals force, and chemical affinities. 

**Figure 1:**
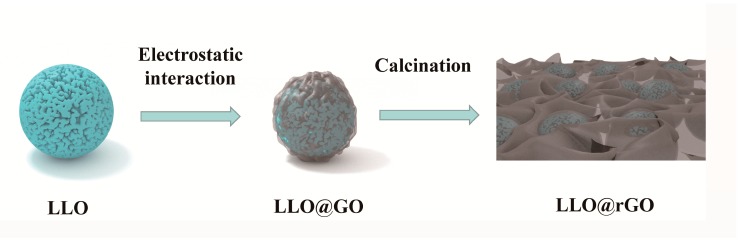
Schematic diagram of the preparation of modified LLO (LLO@rGO), where LLO is layered lithium-rich manganese oxide, and rGO is reduced graphene-oxide.

Therefore we developed a novel and robust method to coat thermally-reduced graphene oxide (GO) uniformly on the LLO surface, mainly based on electrostatic forces. Specifically, LLO (0.4Li_2_MnO_3_∙0.6LiNi_1/3_Co_1/3_Mn_1/3_O_2_) was synthesized by using a simple and feasible carbonate co-precipitation method in our work. Then, the LLO surface was modified with an alkyl-bromide surfactant, which has been successfully applied in other fields [26,27] to make the surface more positive. Thus, a uniform coating of GO on the LLO surface (LLO@GO) has been achieved. Finally, the GO-coated LLO composite was annealed to obtain rGO-coated LLO (LLO@rGO) particles for high-performance lithium-ion batteries (LIBs). The schematic diagram of the preparation of LLO@rGO is shown in the Figure 1. We found that the rGO coating can greatly improve the cycling and rate performance of lithium-rich cathode materials. 

## 2. Results and Discussion

**Figure 2:**
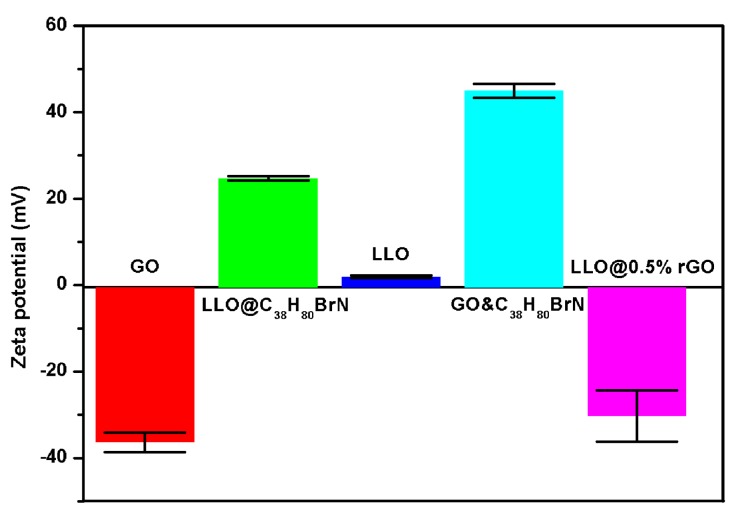
Potentials of pristine layered lithium-rich manganese oxide (LLO), LLO modified with a surfactant, and graphene-oxide (GO) sheets in aqueous solutions. Dimethyldioctadecylammonium bromide was used as the surfactant.

To verify the working mechanism of the surfactant, the zeta potentials of pristine LLO, surfactant-modified LLO in aqueous solutions, were measured as shown in Figure 2. It can be seen that the zeta potential of the pristine LLO is between 0 and 5 mV, which is nearly neutral. Meanwhile, LLO modified with a surfactant, dimethyldioctadecylammonium bromide (C_38_H_80_BrN), shows a far higher positive potential. Obviously, the surfactant greatly changes the surface charge state. Such a phenomenon is due to a bilayer surfactant structure formed on the LLO surface. Previous study showed that the alkyl-bromide surfactant can be adsorbed on a cathode oxide based upon the interaction between the cationic heads of the surfactant, and the surface O of the cathode oxide [28]. Thus, in the inner layer close to the LLO surface, surfactant molecules are adsorbed on the LLO surface with the long alkyl tails far away from the LLO surface. Meanwhile, in the outer layer, the long alkyl tails of the surfactant molecules will interact with their counter parts in the inner layer and the cationic heads of the surfactant molecules are left outside [29]. 

Hence, the final charged state of the surfactant-modified LLO will be determined by the outside cationic heads of the out-layer surfactant, and it is highly positive [30], which can make the modified LLO to be easily dispersed in aqueous solutions for further GO coatings [31]. 

To confirm the strong electrostatic interaction between the GO sheets and the surfactant, their zeta potentials were also measured. The zeta potential of GO sheets is presented in Figure 2, which shows a widely-known negative potential, while the C_38_H_80_BrN-GO mixture shows a positive potential. This is because the negatively-charged GO sheets are fully covered by the surfactant molecules, and are reversed to a positively-charged state. The results prove the strong interaction between the surfactant and the GO via the electrostatic interaction. No doubt, GO will coat tightly on the surfactant-modified LLO surface when they are mixed. Moreover, after the GO coating, the surface state of the LLO@rGO particles should be dominated by the coating nanosheets, which is negative, as shown in Figure 2.

**Figure 3:**
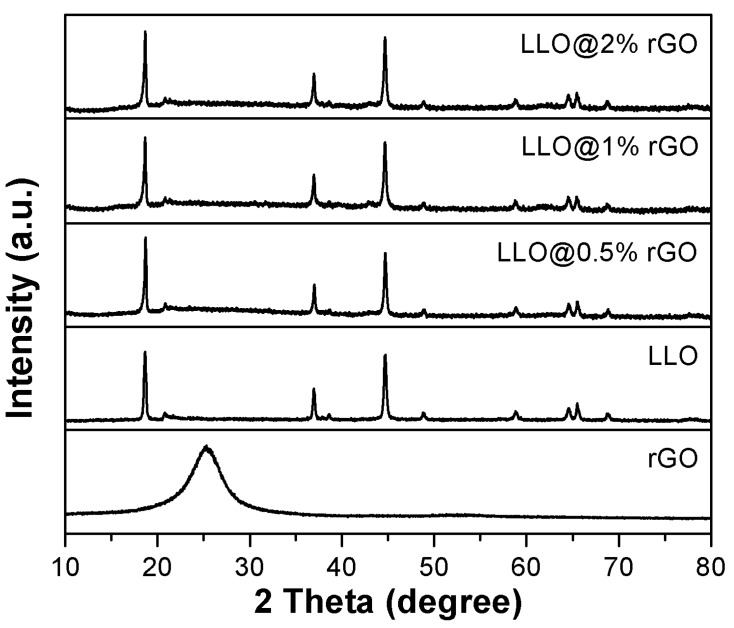
Patterns of reduced graphene-oxide (rGO), LLO, the as-prepared LLO@rGO with different amounts of rGO.

The effect of rGO coating on the crystal phase evolution of LLO was first determined. Figure 3 shows the X-ray diffraction (XRD) patterns of rGO, LLO and LLO@rGO powders with different coating amounts of rGO. The strong diffraction peaks of LLO and LLO@rGO can be indexed to a layered hexagonal LiMO_2_ (R3m) phase, a typical α-NaFeO_2_-type structure. The weak peaks at 20–25° belong to a monoclinic Li_2_MnO_3_ (C2/m) phase [32,33,34]. Besides, the separations of the (006)/(102) (at 37.6–39°) and (108)/(110) (at 64–66°) peaks, are assigned to the formation of a preferable ordered hexagonal-layered structure [35,36]. These results are consistent with those reported in the literature. For the pure rGO sample, the featural peak at 25.2° indicates the complete reduction of the GO sheets [37]. However, no featural peak belonging to the rGO can be observed from the different LLO samples. It might be due to the tiny quantity of the rGO coatings on the LLO surface. Nevertheless, the XRD results indicate that the rGO coating with the aid of the surfactant have no effect on the LLO phase structure, which is quite important for keeping the instinct nature of LLO material.

**Figure 4:**
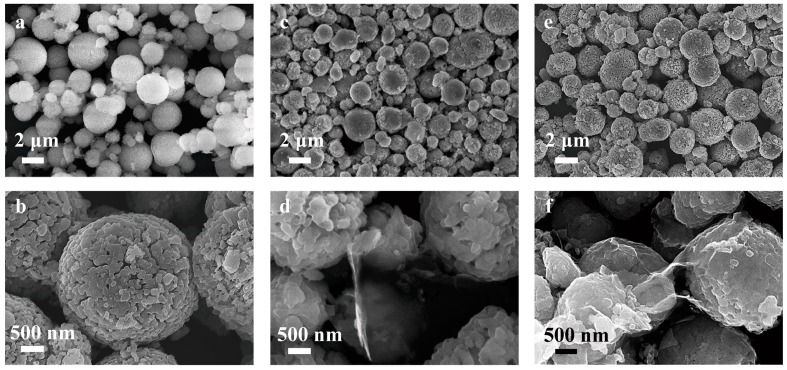
Images of (**a**,**b**) pristine LLO powders, (**c**,**d**) rGO-coated LLO powders without the use of the surfactant and (**e**,**f**) the LLO@rGO powders. The weight ratio of rGO to LLO is 0.5%.

Next, the effect of rGO coating on the morphology of LLO were also checked to see if the rGO nanosheets could wrap the LLO particles well, as shown in Figure 4. For comparison, the rGO-coated LLO without the use of the surfactant was also observed. It can be seen that the pristine LLO powders show a 3D micro-sphere shape with diameters of 2–4 µm which are composed of nanoparticles, as shown in Figure 4a,b. When rGO was added without using the surfactant, rGO are preferentially existing between LLO particles, as shown in Figure 4c,d. While the surfactant was used to tune the surface potential of LLO powders, rGO sheets would wrap the LLO surface tightly, as shown in Figure 4e,f. Hence, we believed that with the aid of the surfactant the LLO cathode material with rGO coating would exhibit outstanding performance, which is presented in the following text. 

**Figure 5:**
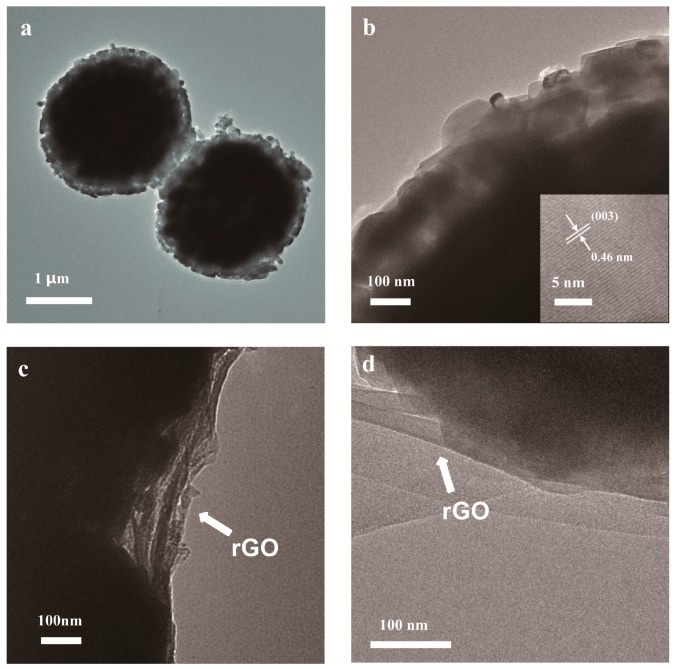
Patterns of (**a**,**b**) pristine LLO, (**c**,**d**) LLO@rGO.The weight ratio of rGO to LLO is 0.5%.

To further exam the details of the morphology of rGO-coated LLO, transmission electron microscopy (TEM) images of LLO and LLO@rGO are shown in Figure 5. It can be seen that there are some voids on the LLO powder surface, as shown in Figure 5a. As we know, the pristine LLO powders are composed of nanoparticles, which can lead to the rough surface, and are clearly consistent with the scanning electron microscope (SEM) images in Figure 4b. To confirm this, the high-resolution TEM (HR-TEM) image was taken from the edge of the lateral plane of the pristine LLO (Figure 5b). The clear lattice fringe is calculated to be 0.46 nm, which can be assigned to both the (003) plane of the rhombohedral phase and the (001) plane of the monoclinic phase [38]. When the rGO sheets were coated on the LLO surface, the voids on the powder surface will be covered by the rGO sheets as shown in Figure 5c,d. 

It is obvious that the rGO wraps the LLO particles tightly, owing to the addition of the surfactant. We believed that such kind of structure is beneficial for the improvement of the electrochemical properties of the LLO material.

**Figure 6:**
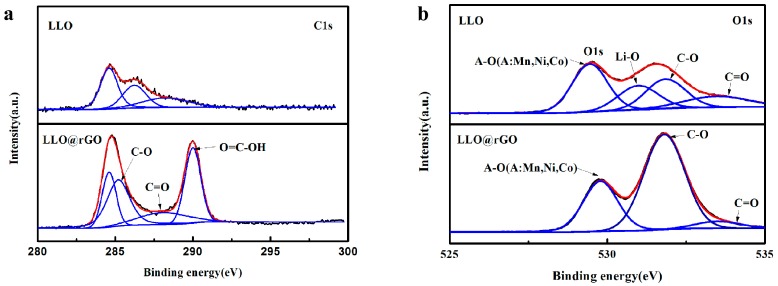
Resolution X-ray photoelectron spectroscopy (XPS) spectra of (**a**) C1s and (**b**) O1s of LLO and LLO@0.5%rGO.

X-ray photoelectron spectroscopy (XPS) was used to monitor the evolution of the chemical states of the particle surface to further ensure the coating of rGO. Figure 6a shows the XPS spectra of C1s, O1s orbitals of LLO and LLO@0.5%rGO, respectively. The features of the C1s spectrum of the bared LLO are similar with respect to the previous report [39]. With the coating of rGO nanosheets, the bonding energies of 285.2, 288.1 and 289.9 eV for the LLO@rGO sample, which belong to the C–OH, C=O and O=C–OH functional groups of rGO sheets [40], are much stronger than those of the pristine LLO. The features of the O1s spectrum of the pristine LLO are also consistent with the previous report [41]. When the pristine LLO was coated with rGO nanosheets, a strong bonding energy of 531.8 eV, which is assigned to the C-OH functional groups of rGO sheets [39], is much stronger than that of the pristine sample. The XPS results also confirmed the existence of rGO nanosheets on the LLO surface. 

**Figure 7:**
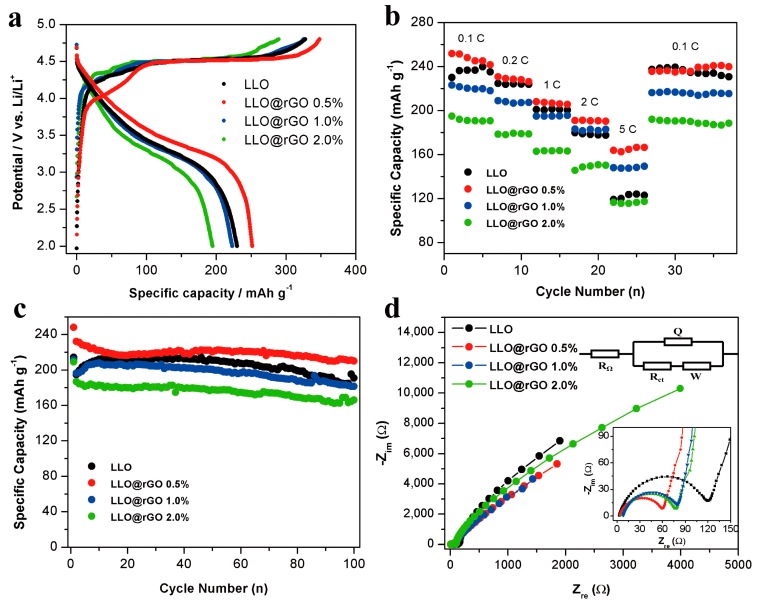
Electrochemical performances of the as-prepared LLO@rGO electrodes with different amounts of rGO: (**a**) The initial charge and discharge profiles at 0.1C, (**b**) rate capability, (**c**) cycling stability, and (**d**) EIS plots, and the corresponding fitting using an equivalent circuit.

Electrochemical properties of the modified LLOs and pristine LLO were side-by-side compared to determine the effect of rGO coatings with the aid of the surfactant. The first cycle voltage profiles of LLO and LLO@rGO electrodes obtained at 0.1C are shown in Figure 7a. The Li^+^ ion is extracted from the active LiMO_2_(M=Ni, Co, Mn) phase, since the voltage gradually increases up to 4.50 V from 4.45 V, accompanied by the oxidation of Ni^2+^ to Ni^4+^ and Co^3+^ to Co^4+^. Then, the voltage maintains a long plateau at 4.50 V, during which Li^+^ ions are further removed from the Li_2_MnO_3_ component (TM layer) [42]. Figure 7a indicates that the capability of LLO@0.5%rGO is higher than those of the pristine LLO, LLO@1.0%rGO, LLO@2.0% rGO. It can be concluded that a proper amount of rGO is required for LLO modification. Over-coated rGO will impair the capacity because of the hindered Li^+^ ion transport.

To further elucidate the changed electrochemical performance of the LLO by the rGO modification, extended rate capability testing was implemented, as presented in Figure 7b. The pristine sample shows poor values at high rates. However, the LLO@0.5%rGO electrode delivers a discharge capacity of 166.3 mAh g^−1^ at the 5C rate, which is far higher than that of the LLO electrode (122.8 mAh g^−1^). This high-rate capability is attributed to the highly conductive ultrathin graphene (0.5%) around LLO particles. Moreover, the rGO coatings can also reduce the electrolyte decomposition and serve as a fast pathway for electron transportation during the charge/discharge process [43,44]. Meanwhile, rGO-modified LLO exhibits excellent capacity reversibility. The discharge capability returns to 241 mAh g^−1^ when the discharge rate is back to 0.1 C. Cycling stability is also an important issue for high-performance materials. Figure 7c shows the cycling performances of pristine LLO and LLO@rGO at 0.2 C. 

It can be seen that the discharge capacity of the pristine-LLO electrode is going to decay from 40 cycles. Surprisingly, the LLO@rGO electrode manifests a stable capacity retention after 100 cycles. The improved cycling capacity is owed to the physical protection layer provided by a proper amount of rGO wrapping layers. The rGO layer can separate active material and electrolyte away, while suppressing the attack from HF and inhibiting the surface side reaction [45,46]. 

To clarify the kinetic features of pristine-LLO and LLO@rGO-based cells, EIS measurement was also performed. As shown in Figure 7d, the semicircle at the high-frequency region is ascribed to the charge transfer resistance. The straight line at the low frequency region is attributed to the solid phase diffusion process. To scrutinize the effect of rGO on the electrochemical behavior, an equivalent electrical circuit model was used to extract the electron and ion transport parameters, as shown in the inset of Figure 7d. R_s_, R_ct_, Q and W represent the ohmic resistance of the electrode, charge transfer resistance at the electrode/electrolyte interface, double layer capacitance, and Warburg impedance of the Li^+^ diffusion in the electrode, respectively. It can be seen that the values of R_s_ of various cells are almost the same. R_ct_ shows big difference in different cells. The LLO@rGO-0.5%-based cell displays the lowest value of R_ct_ compared with those of other cells, as shown in Table 1. A previous study indicated that the conductive rGO on the cathode material is beneficial for the interface charge interaction to reduce the interfacial transfer. [47] In our study, the surfactant-assisted coating rGO is well-wrapped on the LLO surface, which attributed to the greatly reduced Rct. Furthermore, calculated diffusion coefficients of the Li ion within the different LLO particles show that the coating rGO affects the solid state diffusion, as shown in Table 1. It can be seen that the coating of rGO nanosheets can enhance the Li ion diffusion within the solid-state phase, owing to their excellent electronic conductivity [47]. 

## 3. Materials and Methods 

### 3.1. Synthesis of 0.4Li_2_MnO_3_∙0.6LiNi_1/3_Co_1/3_Mn_1/3_O_2_ (LLO)

The layered lithium-rich manganese oxide (LLO) cathode material was synthesized by a carbonated co-precipitation method. An aqueous solution of NiSO_4_·6H_2_O, MnSO_4_·H_2_O and CoSO_4_·7H_2_O (Ni:Co:Mn = 0.6:0.2:0.2) was stirred at room temperature to achieve a precursor solution. Then, an adequate amount of precipitant (NH_4_HCO_3_) was added to the above solution, which was kept stirring at room temperature for 2 h. Next, the solution was filtered, washed and dried. The dried powder was mixed with LiOH·H_2_O, and then calcined at 400 °C for 2 h, 600 °C for two more hours, and 800 °C for a final five hours in air, to obtain the pristine LLO.

### 3.2. Synthesis of modified LLO (LLO@rGO) 

GO was prepared by the Hummer’s method. To wrap reduced graphene-oxide (rGO) onto the LLO surface, 0.15 g LLO powders was first dispersed in 10 mL deionized water. Then, 3 mg dimethyldioctadecylammonium bromide (C_38_H_80_BrN) was added into the LLO solution under continuous stirring. Next, different amounts of GO solution (12 mg/mL) were added to the modified-LLO solution at different weight ratios (GO to modified LLO: 0.5 wt%; 1 wt%; 2 wt%), and were stirred at 60 °C overnight. The mixed solutions were centrifuged and then dried at 60 °C for 12 h. Finally, the as-prepared powders were annealed at 400 °C for 5 h in an Ar atmosphere to obtain the LLO@0.5% rGO, LLO@1.0% rGO and LLO@2.0% rGO.

### 3.3. Characterization

X-ray diffraction (XRD) patterns of the samples were obtained by a Rigaku D/max 2500 diffractometer with Cu Kα radiation at V = 4 0 kV and I = 150 mA in a 2θ range of 10–80°, and the scanning rate was 10° min^−1^. The electronic states of the elements were analyzed with a K-Alpha X-ray photoelectron spectroscope (XPS) (ESCALAB 250Xi) (Thermo Fisher Scientific, Waltham, MA, USA). Morphological observations were carried out on a Zeiss Supra 55 field emission scanning electron microscope (FE-SEM) (Carl Zeiss AG, Oberkochen, Germany). The transmission electron microscopy (TEM) and high-resolution transmission electron microscopy (HR-TEM) were used for morphology and micro-crystal observation with a JEM-2100 transmission electron microscope (JEOL, Tokyo, Japan). The weight contents of rGO in the LLO@rGO composites were determined using thermo-gravimetric analysis (DSC-TGA, SDT-Q600) (TA Instruments, New Castle, DE, USA).

### 3.4. Electrochemical Measurement

All the electrochemical tests here were carried out on CR2032-type coin cells with lithium metal as their anodes. The details are described as follows. A slurry, with 75 w% active materials, 15 w% acetylene black, and 10 w% polyvinylidene fluoride dispersed in *N*-methyl-2-pyrrolidone, was prepared and then coated on an aluminum foil to fabricate working electrodes. The cells were assembled in an Ar-filled glove box with water and oxygen contents less than 0.1 ppm, using polypropylene film (Celgard 2400) as the separator. The electrolyte was a solution of 1 M LiPF_6_ in a 1:1 (*v*/*v*) mixture of ethylene carbonate (EC)/dimethyl carbonate (DMC). The cells were galvanostatically charge-discharged at various current rates (1C = 250 mA g^−1^) between 2.0 and 4.8 V at room temperature. The cyclic voltammetry (CV) tests were conducted between 2.0 V and 4.8 V at a scan rate of 0.1 mV s^−1^ on an electrochemical workstation. Electrochemical impedance spectroscopy (EIS) was also performed on an electrochemical workstation (Chenhua 660D, China). The frequency of EIS ranged from 100 kHz to 10 mHz with an amplitude of 5 mV.

## 4. Conclusions

In conclusion, we have successfully fabricated a high performance rGO-coated LLO cathode material. A surfactant was proved to be beneficial for the robust coating of rGO nanosheets on the LLO powder surface. The rGO-coated LLO cathode material exhibited outstanding rate capability as 166.3 mAh g^−1^ at 5C rate. The results show that the rGO coating enables a greatly enhanced rate performance as well. Such improvement is due to the enhanced interfacial charge transfer, the reduced side reactions and the inhibited structural deterioration of Li-rich cathode materials. Using a proper surfactant to enhance the coating of graphene derivatives on different surfaces is demonstrated to be very promising to realize outstanding energy storage devices. 

## Figures and Tables

**Table 1:** The fitting values of the components in the simplified equivalent circuit.

Components	R_Ω_/Ω	R_ct_/Ω	R_total_/Ω	Diffusion Coefficient/ 10^−17^cm^2^s^−1^
LLO	3.01	113.50	116.51	2.21
LLO@rGO 0.5%	3.73	57.11	60.84	6.22
LLO@rGO 1.0%	6.58	72.22	78.80	4.22
LLO@rGO 2.0%	8.04	72.87	80.91	5.21
